# Conceptualizing Community Mobilization for HIV Prevention: Implications for HIV Prevention Programming in the African Context 

**DOI:** 10.1371/journal.pone.0078208

**Published:** 2013-10-11

**Authors:** Sheri A. Lippman, Suzanne Maman, Catherine MacPhail, Rhian Twine, Dean Peacock, Kathleen Kahn, Audrey Pettifor

**Affiliations:** 1 Center for AIDS Prevention Studies, Department of Medicine, University of California, San Francisco, San Francisco, California, United States of America; 2 Gillings School of Global Public Health, University of North Carolina at Chapel Hill, Chapel Hill, North Carolina, United States of America; 3 Wits Reproductive Health and HIV Institute (WRHI), School of Clinical Medicine, Faculty of Health Sciences, University of the Witwatersrand, Johannesburg, South Africa; 4 Collaborative Research Network, University of New England, Armidale, New South Wales, Australia; 5 Medical Research Council/Wits Rural Public Health and Health Transitions Research Unit (Agincourt), School of Public Health, Faculty of Health Sciences, University of the Witwatersrand, Johannesburg, South Africa; 6 Sonke Gender Justice Network, Cape Town, South Africa; The University of New South Wales, Australia

## Abstract

**Introduction:**

Community mobilizing strategies are essential to health promotion and uptake of HIV prevention. However, there has been little conceptual work conducted to establish the core components of community mobilization, which are needed to guide HIV prevention programming and evaluation.

**Objectives:**

We aimed to identify the key domains of community mobilization (CM) essential to change health outcomes or behaviors, and to determine whether these hypothesized CM domains were relevant to a rural South African setting.

**Method:**

We studied social movements and community capacity, empowerment and development literatures, assessing common elements needed to operationalize HIV programs at a community level. After synthesizing these elements into six essential CM domains, we explored the salience of these CM domains qualitatively, through analysis of 10 key informant in-depth-interviews and seven focus groups in three villages in Bushbuckridge.

**Results:**

CM domains include: 1) shared concerns, 2) critical consciousness, 3) organizational structures/networks, 4) leadership (individual and/or institutional), 5) collective activities/actions, and 6) social cohesion. Qualitative data indicated that the proposed domains tapped into theoretically consistent constructs comprising aspects of CM processes. Some domains, extracted from largely Western theory, required little adaptation for the South African context; others translated less effortlessly. For example, critical consciousness to collectively question and resolve community challenges functioned as expected. However, organizations/networks, while essential, operated differently than originally hypothesized - not through formal organizations, but through diffuse family networks.

**Conclusions:**

To date, few community mobilizing efforts in HIV prevention have clearly defined the meaning and domains of CM prior to intervention design. We distilled six CM domains from the literature; all were pertinent to mobilization in rural South Africa. While some adaptation of specific domains is required, they provide an extremely valuable organizational tool to guide CM programming and evaluation of critically needed mobilizing initiatives in Southern Africa.

## Introduction: Community Mobilization for HIV Prevention

Community mobilizing strategies, designed to engage and galvanize community members to take action towards achieving a common goal [1], are increasingly recognized as essential components of HIV prevention programs. In the area of HIV prevention, community mobilizing interventions have demonstrated successes in increasing condom use [2-7], improving service access and quality [7,8], increasing social capital or social cohesion [7,9] and most recently in promoting uptake of HIV counseling and testing [10]. Beyond these demonstrated successes, community mobilization (CM) will play a key role in effective implementation of key bio-medical interventions in the future. For example, landmark trials have demonstrated the efficacy of early antiretroviral treatment (ART) for HIV positive individuals to prevent transmission to uninfected partners [11] and providing ARTs to high risk HIV negative individuals to prevent acquisition of the virus [12]. The success of treatment-as-prevention approaches hinges on developing CM strategies to inspire broad support for care and treatment for those living with or at elevated risk for HIV/AIDS, their providers, and their family and community networks. Unleashing the potential of community mobilization for HIV prevention is particularly critical in sub-Saharan Africa, which shoulders 70% of the global HIV epidemic [13]. 

Community mobilization interventions to prevent HIV have varied widely in tactics and focus. A number of CM efforts have included components that address the larger social and structural context surrounding HIV, including efforts to reduce discrimination against groups most vulnerable to HIV; to create social cohesion and extend social networks for disenfranchised communities; and to ensure community participation in prevention and care programming [14-17]. The best known HIV prevention mobilizing effort was undertaken by sex workers in Sonagachi (Kolkata), India. Over 15 years of evolving participatory prevention and organizing, condom use increased and remained high and HIV prevalence declined and remained low among sex workers in Kolkata as compared to sex workers in other Indian cities [3]. The basic components of the Sonagachi project included the establishment of quality STI/HIV testing and treatment clinics; facilitated access to condoms; training of peer outreach workers, who over time became leaders for collective action; political advocacy and formation of broad partnerships; founding of a literacy program and a loan service program at a credit union; and the establishment of a collective organization[3,18,19]. While the Sonagachi initiative evolved into a community-led program, constantly adapting to respond to local needs and opportunities, other mobilizing projects have included community participation but largely remained externally run interventions with prescribed components. Project ACCEPT (HPTN 043), for example, was an NIH funded multi-site randomized community trial that aimed to change community norms and reduce risk for HIV infection through community-based HIV counseling and testing, community outreach, and post-test support [10]. The seven major community mobilization (outreach) strategies used in Project Accept included: forging stakeholder buy-in, formation of community coalitions, community engagement, community participation, raising community awareness, involvement of leaders, and partnership building [20]. Results from ACCEPT indicate that mobilizing communities around HIV testing drastically improved testing uptake and may have lowered the rate of new HIV infections, particularly among women 24-35 years of age [21]. The experiences in Sonagachi and ACCEPT represent examples of successful mobilization initiatives in practice, yet, there are many efforts that apply the term “community mobilization” rather arbitrarily to describe activities ranging from peer education to conducting social media campaigns. Evans and colleagues have argued that “activities that target and aim to empower individual community members should be distinguished from community mobilization efforts which seek to construct a collective entity out of a group of individuals.”[17] Most mobilizing projects have failed to make this distinction or explicitly focus on community change; others fail to describe what the process of mobilization around HIV prevention entails; fewer still have elaborated on the underlying theory guiding the selection of activities. 

Currently there is widespread buy-in for community mobilization [22] and yet very little published work that lays a conceptual foundation for how to mobilize communities around HIV prevention and no consensus around the core components of mobilizing [20,23]. Most practical guides for CM in public health (including, but not limited to HIV prevention) come from larger international institutions’ resources for community-based work, including publications from UNAIDS, WHO, and CDC. Some resource guides are quite substantial [24], others are helpful in that they lay out recommendations for activities [1,25,26]; however they are also largely atheoretical and pay little attention to contextual factors that play a large role in successes and failures [18]. Overall, explicit theory-based practice in CM work is largely missing, despite the fact that a clear conceptual framework and identification of core CM components would benefit the field enormously. Fortunately, there is a great wealth of relevant but under-utilized sociological, community empowerment, community development, and community capacity literature on which to build stronger theory and thus a framework to guide CM programming. A more nuanced conceptual understanding of community mobilization (CM), including further conceptual development of the dimensions or core components of mobilization for HIV prevention, will improve design, implementation and evaluation of mobilizing interventions across environments [20]. 

We undertook a two-step process in order to lay a strong theoretical foundation for community mobilizing efforts. First, we carried out an extensive literature review of relevant social science theory in order to identify potential elements of CM. We then extracted six domains of mobilization that we hypothesized were necessary to improve health outcomes or behaviors. We present a synthesis of the literature, summarizing relevant theory from each contributing discipline and highlighting common elements across disciplines that can be addressed by health promotion or intervention programs. Second, we conducted qualitative research to explore the relevance and value of the CM domains, based largely on Western theory, in the context of HIV prevention in rural, sub-Saharan Africa, where mobilization programs are critically needed. These two theoretical steps presented in this manuscript, aim to move the field closer to the ultimate goal of developing a strong theory-based framework for future CM programming for HIV prevention; including CM work being undertaken as part of a large HIV prevention trial underway in rural South Africa.

## Methods

### Literature Review

We explored literature from four general disciplines; social movements (sociology), community empowerment, community development, and community capacity, including review of both theoretical texts and applied work. We aimed to identify universal concepts that are both relevant to health promotion and that can be modified through programming. The review started with the strongest theoretical framework – the vast field of social movements literature, which itself includes multiple strains of thought. While social movement theory generally focuses on understanding the movements of nations, classes, races, and large scale change in society, we sought elements relevant to community mobilization, envisioning CM as a mezzo-level ancillary to macro-level social movements. The social movements review was conducted utilizing the main texts from the most prominent social movement theorists and included creating an outline of the most consistent key principles and elements that precede change in a community or movement. Noted CM domains or potential key candidates were defined and placed in a spreadsheet for comparison with other disciplines. We then reviewed the fields of community empowerment, community organization or development, and community capacity building. These overlapping and complementary literatures are directly relevant to interventions that aim to mobilize communities to forge healthier environments. The key domains of these literatures were also placed in the spreadsheet to note commonalities with each other and social movements literature. Comparison of key elements across literatures resulted in the extraction of six proposed domains. In Table 1 we summarize how each of the reviewed literatures addresses or frames the hypothesized domains of community mobilization (each row represents a proposed domain and framing across disciplines).

**Table 1:** Proposed domains of Community Mobilization and their framing in related disciplines.

	**Literatures Reviewed**				
**Proposed Domains of Mobilization**	**Public health / programmatic literature**	**Social movements**	**Community empowerment**	**Community organization / development**	**Community Capacity**
**Shared concern**	Programming target	Collective claims / defined “opponent”	Problem assessment	Issue selection	Shared values / purpose / norms
**Community consciousness**	Raising consciousness	Framing / cognitive liberation	Asking why	Critical consciousness	Learning culture / critical consciousness
**Organizational structure / networks**	Building coalitions and organizational links	Mobilizing structures / Informal exchange / Networks & coalitions	Organizational structure / Links to others	Community capacity (ability to mobilize – includes organizational resources)	Structures (social and inter-organizational networks & community spaces)
**Leadership**	*assumed someone is leading the effort - often includes training	Movement leaders / entrepreneurs	Leadership	Community capacity (ability to mobilize – includes human resources)	Leadership (also includes resources such as human capital)
**Collective actions**	Taking action together	Movement repertoire (public meetings, rallies, numbers)	Participation	Participation	Civic participation
**Social cohesion**	*not addressed*	Collective identity	Building community trust	Building sense of community	Social relationships (social connectedness, trust, sense of community)

* Note: While the role of outside agents (community empowerment) can be considered a resource and political opportunity (social movements), the presence of an outside group seeking partnerships, bringing funding, or providing technical assistance is implicit in the community health and HIV prevention framework, and is not included as a domain of community mobilization.

### Qualitative Study Setting

Because it is unknown how well these hypothesized CM domains, gleaned largely from Western theories, apply to the sub-Saharan African context, we explored the relevance of these CM domains using qualitative data collected in a rural, South African community. Qualitative data was collected in the Agincourt sub-district of Bushbuckridge, Mpumalanga province, about 500 km northeast of Johannesburg, near the border with Mozambique. The area is home to the Agincourt Health and Socio-demographic Surveillance Site (AHDSS) where the MRC/Wits Rural Public Health and Health Transitions Research Unit (Agincourt) runs an annual census of the approximately 90,000 people living in 16,000 households in the 27 contiguous villages of the AHDSS. HIV prevalence is extremely elevated in the area, documented at over 45% among 35–39 year olds in a recent bio-behavioral survey [27]. In order to span a range of socio-demographic characteristics and community resources, participants were recruited from three villages purposively selected on population size, distance to transport hubs, and proportion of Mozambican immigrants. 

The AHDSS is currently the site of a randomized trial (1R01MH087118-01/HPTN 068) which aims to determine the effects of a combined conditional cash transfer (CCT) and community mobilization intervention in reducing HIV infection among young South African women [28]. The community mobilization component of the trial, which was guided by the concepts outlined here, focuses on raising consciousness around the intersection of HIV and gender norms that place young women at risk. 

### Data collection

Two sampling frames were used for participant recruitment. First, key informants or community leaders were identified by the AHDSS “LINC” Office (Learning, Information dissemination and Networking with Community) from members of the village Community Development Forums (CDF), local Ward committees (lowest tier of local government), schools, and other active citizens who LINC has identified over the course of 10 years working in the villages. Using a roster of 30 informants across the 3 villages, ten key informants (four women and six men across three villages) were selected to participate in an individual in-depth-interview. Selection was purposeful to ensure gender and age balance and representation from the public sector, private sector, and civil society. From the remaining 20 key informants on the list, an additional 10 were invited to participate in a FGD of community leaders, again selected to be as representative of diverse interests, ages, and gender as possible. We also recruited 18-35 year old village residents to participate in six mixed-gender community FGDs (two in each village). These participants were randomly selected within gender and socio-economic strata from the AHDSS sampling frame; the sampling frame includes all households in the area, which have been fully enumerated, and is updated each year as part of the census. FGD participants were contacted in person to assess willingness to participate. 

Overall 64 people participated in qualitative data collection, which was conducted in the local language - Xitsonga (Shangaan). Each participant provided written informed consent prior to commencing participation. All consent and ethical procedures were approved by the Human Research Ethics Committee at the University of the Witwatersrand, the Committee for Human Research at the University of California, San Francisco, and the Institutional Review Board at the University of North Carolina, Chapel Hill. 

The semi-structured FGD and IDI guides aimed to elicit local perceptions and concepts of mobilization (introduced as “working together to solve community problems”) and were organized around the hypothesized CM domains. Topics in the guides included shared concerns; critical consciousness; leadership; organizational structures and resources, including presence of community based organizations (CBOs); and community experiences in collective action, including experiences working towards shared goals, and community processes when issues of concern arise. While social cohesion was not included as a separate topical area in the guide, responses regarding collective action and how / why the community does or does not work together were replete with statements regarding the presence or absence of shared identities; much of the results section below regarding social cohesion was garnered from those statements. The interviewers were trained to utilize the guides in eliciting specific information, but also to explore emerging issues. 

### Data management and analysis

All interviews and focus groups were digitally recorded and simultaneously translated and transcribed into English by the fieldworker who had facilitated the focus groups. Analysis was first organized around the hypothesized domains of mobilization. The data were initially coded deductively using codes that represented the topics covered in the data collection. Based on review of the deductively coded data, the authors developed inductive codes to flag data on themes that emerged within or across the topics. Atlas.ti was used to apply the deductive and inductive codes to the data. Code reports were generated and reviewed by SL, SM and AP. Authors discussed and clarified the main ideas that emerged within the different code reports, with a focus on whether the domains resonated and how they appeared to operate in this context, as well as assessing departures from the intentioned meaning of CM domains based on the literature review. The authors also explored relationships within the data among themes to examine how the domains interact and affect community mobilization in this context [29,30]. 

## Results: Conceptualizing Community Mobilization

### Social Movements Theory

Social movement theory has a number of relevant frameworks that illustrate the needed movement components (or core elements) to create change at a community level. Charles Tilly treats social movements as a form of contentious politics and postulates that social movements have three elements: 1) an organized public effort making collective claims on target authorities; 2) social movement repertoire, including creation of associations and coalitions, public meetings, rallies, etc.; and 3) participants’ public representations of worthiness, unity, numbers, and commitment [31]. Within this definition, community movements would need to include a demand- something that requires change, organized actions, and well attended public activities. 

Gaining a dominant position in social movement theory in recent decades is a structural approach, collectively Resource Mobilization and Political Process models, which “explain insurgency on the basis of a favorable confluence of factors internal and external to the movement ”[32]. Specifically, three key ingredients shape collective action. *Political opportunities* are moments when the power structure or political establishment is undergoing change or challenge, and when opposition groups can voice their interests. *Mobilizing structures* are “those collective vehicles, informal as well as formal, through which people mobilize and engage in collective action” [32]; including varied organizational groups or networks. *Framing processes* (or cognitive liberation) are collective interpretation and attribution processes, in which a group collectively defines their position as unjust and amenable to change through group action. 

Other strains of social movements theory focus on the structure and interaction of networks, placing more emphasis on *informal exchanges* between members of networks or organizations engaged in collective projects and *collective identity* between members of the networks than on the actual content of the collective actions themselves [33]. These network theorists depart from earlier conceptualizations of movements in envisioning collective identity as paramount: collective actions are relevant to social movements only when carried out in coordination and exchange between individuals and organizations. Another departure from traditional social movement theory is comprised of the New Social Movement (NSM) theorists. One major divergence is the view of shared goals (claims or conflict): while most social movement theorists maintain that claims need to focus on redistribution of resources, NSM theorists contend that the target of change can focus on social inclusion or consciousness raising in and of itself, which is highly relevant to the health promotion framework in communities [34,35]. 

Among the varied frameworks of social movements, the basic components most relevant to smaller community contexts include: a shared issue or collective goal, the requirement that the shared concern be built from collective sensitization processes or critical consciousness (framing processes), the presence of an organizational structure or mobilizing network (networks / mobilizing structures), some form of visible collective action or people acting together for change, and some collective identity forged in the community. 

### Community Empowerment, Development and Capacity

The fields of community empowerment, community organization or development, and capacity building are directly relevant to interventions that aim to mobilize communities to forge healthier environments, though rarely have these fields been translated into work with HIV in developing countries. Empowerment has been defined as “a social action process by which individuals, communities, and organizations gain mastery over their lives in the context of changing their social and political environment to improve equity and quality of life”[36]. One way community empowerment can occur is by facilitating involvement in community networks where trust is developed and by encouraging community members to work together to advocate for desired change. Researchers seeking to operationalize community empowerment for health promotion have offered a set of domains that enable mobilization towards shared goals, including: participation, leadership, problem assessment, organizational structure, resource mobilization, links to others, ‘asking why’, program management, and the role of outside agents [37]. As in the New Social Movement theory, outcomes for community empowerment include increased sense of community (identity changes) as well as political transformation [38]. 

Conceptual discussion of the process of community organization has emphasized redress of power imbalances, civic participation and building a sense of community [38]. Minkler and Wallerstein have offered five key concepts of community organization: empowerment, critical consciousness (from Freire’s notion of *conscientização*) [39], community capacity (community ability to identify, mobilize, and address problems), issue selection (identifying unifying, winnable and specific targets of change), and participation and relevance (organizing that engages community members as equals) [38]. 

The concept of community capacity, closely linked and sometimes included within community organization and empowerment frameworks, represents a construct that is at once both a process and desired outcome. In a broad-reaching review by Norton and colleagues, they define community capacity in the context of health promotion as “a set of dynamic community traits, resources, and associational patterns that can be brought to bear for community building and community health improvement” [40]. The set of domains of community capacity collected across fields include individual-level and community attributes and are summarized as 1) skills and resources; 2) social relationships, including trust, reciprocity, and sense of community; 3) structures and mechanisms for community dialogue and collective action; 4) leadership; 5) civic participation, or community engagement with public issues; 6) value system, or shared ideals, like equity, and 7) learning culture, or a community’s ability to “think critically and reflect…”[40].

### Intersections and Commonalities – Reaching a Core set of Domains

All of the literatures universally share 5 common threads (Table 1). The first is the need to identify some claim, shared concern, or issue: the deliberate and participatory process of arriving at a collective claim is common to all of the sociological and health promotion approaches to collective action. The second is the concept of critical consciousness, which is at the heart of the community empowerment and organizational literature and is also encapsulated in “problem assessment,” “asking why,” and the learning culture from community capacity literature. This critical consciousness is akin to cognitive liberation in social movement theory. Collective action is a shared characteristic as well – encapsulated in all references to community participation, civic participation, and movement repertoires or collective activities from social movements. Additionally, all fields recognize the need for some basic organizational structure for a community movement to thrive. These mobilizing structures, or vehicles “through which people mobilize and engage in collective action,” [32] in social movements are comparable to structures and mechanisms for community dialogue in the capacity literature and organizational structure in the community empowerment literature. Finally – some sort of “social glue” is common to all literatures: collective identity is a strong component of the social movement network theorists and the concept of social inclusion is essential in New Social Movements theory. The community capacity literature refers to this as “social relationships - including trust, reciprocity, and sense of community,” empowerment literature as “links to others,” and community organization as “building a sense of community.” 

One domain obviously missing from the social movements literature, but key to the community empowerment, development, and capacity literatures is the domain of leadership. This omission reflects the reticence of social movement theorists to attribute too much influence to individuals, though most recognize that movement leaders or “entrepreneurs” are key [35]. In a community context, small networks of visible activists or engaged political leaders may represent a more salient component for mobilization – as such, leadership, be it a coalition, institution, or small group of activists, was included as a CM domain. Another element that was not common or explicitly included in all literatures is that of political opportunities. In social movements, these refer to trends in the political context that shape the success or failure of a community movement. In the community health promotion context, political opportunities might be more akin to the presence of an outside group seeking partnerships, bringing funding, or providing technical assistance – these opportunities are implicit in the HIV prevention framework. As a result we have not included this as a separate element in mobilizing interventions but it is included implicitly in the other domains (i.e. leadership, networks and organizations). 

Given the above intersections, we hypothesized that community mobilization requires the following: The use of collective activities to promote social change around a shared concern by a group, community or network of communities, which includes six components:

1. a defined or shared concern that is the target of change,
2. community sensitization or critical consciousness raising about the issue,
3. an organizational structure with links to groups/networks,
4. leadership (individual and/or institutional),
5. collective or shared activities/actions, and
6. social cohesion, which includes community ties and working trust.

## Results: Examining Mobilization Domains in Context through Qualitative Research

### Shared concerns: “If you have this HIV problem you … stand by yourself…”

Participants were easily able to identify concerns that were shared in the community. Most commonly these related to infrastructure and safety: primarily access to water, electricity, housing, and crime. Participants unanimously agreed that infrastructure concerns affect the entire village and merit community discussion and action. Neither HIV nor gender inequities, including gender-based violence, were raised as shared concerns by the participants unless prompted by the facilitator. When probed, some participants acknowledged concern because HIV kills people and a few because it affects the most able members of the community (young people). However, the vast majority of participants said it was not a shared concern, being neither an issue that people discussed publically nor a topic of complaint from people living with HIV:

“…but most of the people who are walking in the street they seem like they don’t care about it because they are not talking about this issue.” (FGD 2, Participant B)

Participants mentioned that some individuals still did not believe in HIV/AIDS, preferring to say someone has been bewitched. Participants mentioned that secrecy around HIV stems from stigma. The secrecy in these communities was striking:

“… I can’t say that they don’t know that there is HIV because when I look there is no one who doesn’t know that there is HIV… they know [but] they deny it. Even parents sometimes don’t believe that their child might have HIV, so when [one] is sick they will tell you that he/she has been bewitched and then they will hide him/her in the house….” (Interview E)

“To be honest the community won’t do anything [about HIV/AIDS] because it seems like all the community is sick so who will tell anyone.” (FGD 2, Participant B)

While some participants framed HIV as something with broad, community impact; the majority perceived HIV as something that was a private concern. The pervasive sense from the interviews was that HIV is not an issue owned by the community, as were concerns around water or electricity; instead, there was a common understanding that HIV is a personal issue to be handled alone:

“If you have this HIV problem you have to stand by yourself for your sickness.” (FGD 3, Participant F)

### Critical consciousness: “One person cannot solve this [problem] alone…”

South Africans were collectively well rehearsed in the language of rights, which are enshrined in the South African constitution. Participants had an underlying appreciation of their rights to basic needs: food, water, shelter. When these were inadequate, they gave ample examples of contention and demand, centering on requests of the elected leadership to fix infrastructure problems. When government was viewed as remiss in providing services, community discussion regarding the roots of these problems usually centered on corruption; this engenders frustration and at times, defeatism. For example, participants discussed the shortage of employment and RDP homes (the government’s Reconstruction and Development Programme to address housing shortages) and their distribution channels:

“I can say [a problem] is RDP houses. There is favoritism when it comes to delivering houses. If you are not the relative of the person who is responsible to give houses, even if you deserve it, you won’t get it.” (FGD 1, Participant A)

When confronted with injustices, nepotism, or ineffective leaders, participants’ responses were mixed with indignation, narratives of passivity or defeatism, and some kernels of constructive thinking. Unlike most themes explored in this research, this differed between communities: those communities who felt betrayed by their leaders had stopped processing issues and solutions, whereas those who felt heard by their leaders were more empowered to bring about change. Members of communities with some faith in their leaders voiced recognition of power in numbers. In approximately one third of the interviews participants acknowledged that communities that worked together had a better chance of resolving problems. For example, in discussing the issue of water, a participant said:

“One person cannot solve this [water problem] alone, even if he reports it to the [community development] forum that it is broken and he goes to the department and tells them, alone they won’t believe you, that is why it is important to all meet and discuss the next step about water or how we can do it… all the stakeholders, when there are problems, we gather and unite.” (Interview E)

While some participants thought that people could resolve issues together, the majority of solutions to problems reflected short term solutions. Only a few participants acknowledged the underlying causes of social problems, referenced the historical context, structural inequities, or long-term problem solving. These few “kernels” of consciousness included recognition of structural causes of disease, such as poverty, lack of employment, and gender power dynamics:

“What makes this issue [HIV] worse is unemployment - I know that if I have a sugar daddy he will give me anything to help me, so if there is employment, at least we will stay away from sex.” (FGD 2, Participant D)

According to Freire, the principal tool for *conscientização is dialogue. The community acknowledged the importance of formal dialogue as a signal of importance, particularly in official settings. For example, when asked why HIV is not important in their community, a community leader stated*:

“It is because even us as leaders, when we meet, we don’t talk about it.” (Interview C)

Another respondent stated that there were few opportunities for formal dialogue about any issues, instead community members spoke informally. However, there was also a sense that informal dialogue did not necessarily lead to action:

“ … so mostly we talk when we just met by an accident and when we see that it’s [a long] time since we got water, so we never sit down or call a meeting to talk about water, never, so we just talk and never get a response.” (Interview I)

Interestingly, the formalization of dialogue and opportunities to convene community discussions seemed the purview of the power structure: the Community Development Forum (CDF) generally called community meetings. The hierarchical structure of these communities, discussed in the next section, framed the context for mobilization. 

### Leadership & Organizational Structure: “Because they are our leaders… if I have problems I go to them.”

There were several well established formal organizations in the villages that community members universally mentioned, including the CDF, *Indunas* or traditional chiefs and their representative traditional council, and elected ward councilors. The CDF is neither elected nor paid, but set up such that community organizations in each village (including schools, churches, soccer clubs, political organizations, youth organizations, choirs, stokvels or savings clubs, and burial societies) have the opportunity to nominate members to the forum. The forums, which had about 7 people in most villages, were charged with hearing community issues and representing village concerns to elected leaders. Respondents also regarded the CPF (community policing forum), a local group dealing with violence and crime, and the ruling party, the ANC (African National Congress), as important leadership structures. 

While these organizations were identified with remarkable consistency as the structures that are in place to address community level problems, the delineation of responsibility among the different organizations was not entirely clear, nor was the manner in which these bodies were selected. Generally for issues related to community development (water, housing and electricity) the community turned to the CDF. For land issues and social problems, community members turned to the *Indunas*, though, in some cases the *Indunas* were also consulted for legal problems or issues of safety, which could also be handled by the CPF. In general, formal channels were adhered to:

Facilitator: “… when you have [problems] as leaders of the communities, what will you do about it?”

Participant: “Look for the problem that happened and which structure it is under, which [structure] can be able to solve that problem because it’s not all the problems that can be solved by all the structures… So if it’s a problem that needs to be solved by the CDF it means it will go to the CDF to be solved, so if it needs to be solved by the Indunas, it will go to them.” (FGD 7, Participant G)

Though community members easily identified formal community leadership organizations, they had trouble identifying informal organizations and networks. When asked about other important groups, organizations or leaders working in the villages, the respondents were mostly silent. When probed in the context of specific problems (e.g. orphans or people with AIDS), people mentioned school groups, funeral societies, youth groups, HIV/AIDS prevention or palliative care groups, as well as church and inter-faith organizations. It was unclear from these data how common these organizations were in the different communities and whether they were understood to play an important function. Also, unlike the formal political structures that had broad oversight on community concerns, these informal organizations tended to stay within the boundaries of their original purpose (i.e. school) and did not facilitate additional collective thinking or action around broader issues in the community. Instead, several people talked about the barriers to the development of organizations within their communities:

“Mostly people look for their own benefit, which is why they don’t work with any organizations. They think if they do [work with organizations] their [own] things will fall apart.” (Interview C)

Family consistently came up in the interviews as the strongest and most dependable social network in this area; however, references to family networks were made in relation to personal support and not explored in terms of potential to address collective concerns in this research. 

Leadership within the community was explored as a separate topic in these interviews and there was direct overlap: identified leaders were always members of formal organizations in the communities. Participants pointed out that adherence to the prescribed approaches for making complaints and seeking solutions from leaders was important; any attempts to circumscribe the power structure was viewed negatively. As a result, while they universally recognized these individuals as leaders and the appropriate people to turn to for community issues, they often did not trust these same individuals and often felt let down by them.

Facilitator: “If you had a problem or question, who do you turn to or trust?”

Participant: “I can go to the Counselor or CDF... To be honest we don’t trust them because they don’t work for the community, but because they are our leaders it is a must that if I have problems I go to them.” (FGD 1, Participant A)

### Collective Action: “We only hold meeting[s]; if it doesn’t work, we leave it like that.”

Community collective action to resolve shared concerns generally adhered to the channels dictated by the CDF and traditional leaders. The first line of action was reporting problems or concerns to the CDF or to an *Induna* at a community meeting; alternatively if a leader was made aware of a problem, they could call a meeting and invite community members. Whether lay community members could initiate a meeting to discuss a problem, however, was questionable. While respondents in the community with the most responsive and inclusive leadership said they had meetings and were often successful in resolving issues, respondents from communities with less responsive leadership were unsure about the utility and the repercussions of calling a meeting.

“We won’t hold meetings, you know my sister what it is like, you can’t call a meeting. Say you want to meet with community members, the meeting had to be called by the community leaders you see, so if you hold a meeting by yourself it will look like you want to gossip about the community leaders meanwhile you want to talk about things that will help the community.” (FGD 2, Participant B)

Outside of meetings with leadership, most respondents felt there were few alternatives to register complaints or seek change. Villagers understood that they were not supposed to circumvent the leadership structures with demonstrations, and the leaders who were interviewed confirmed that perspective. Many believed that community members would look down upon strikes, marches, rallies, or protest actions, and that there could be repercussions for such actions. In fact, working outside of formal channels was complicated by the need for official permission for marches.

“People from here don’t like things like strikes; they don’t like the shame

Mostly they are scared that those who will organize will get in trouble.” (Interview B)

“If we go there with a crowd, the municipality will call the police to come and arrest us because you can’t just march without a permission to march, nowadays when you want to march you have to apply… ” (Interview I)

Despite the disincentive to march or rally, a handful of respondents did cite past instances of *toyi toyi* (protest dance), gatherings to demonstrate support for a victim’s family following a violent crime, and a strike at a local hospital. Additionally, because positive change seemed to depend to a large extent on local leadership, a few respondents suggested entering politics themselves or removing unresponsive or corrupt leaders to improve their circumstances, though that tactic did not seem to have met with great success.

“When we want to resolve the issues in our community we can change the Indunas because they are not working accurately.” (FGD 4, Participant A)

Largely it seemed that varying levels of competence and effectiveness of formal leadership shaped the extent to which people feel disenfranchised and whether they could envision solutions to social change. Some respondents did mention meetings where infrastructure concerns were raised, leaders were engaged, and changes (e.g. installation of water pipes) occurred, but a large majority of respondents felt less optimistic:

“Talk won’t solve a problem; we will only solve it when we meet with the municipality but if we hold meetings it will end up on air. The problem of streets [paving] remains.” (FGD 4, Participant A)

At the same time that collective action, as envisioned in the Western sense, seemed stymied by social, cultural and political regulation, there were many examples of people supporting each other in times of great need. These supportive community activities were not recognized as examples of “action to solve shared concerns” as it was phrased in the interviews, but certainly represented community strategies to jointly resolve needs. Most examples of collective “action” we encountered did not revolve around contentious issues or gaining goods and services that government should provide, but included community responsibilities to support one another. These included: contributing money to families following a death, participation in community credit/loan programs, and, to a lesser extent, political participation (voting). 

### Social Cohesion and Control: “The family with crisis called … and the community members did support them”

While we expected to find broad reports of collective village identities and community cohesion, we instead found a mixed sense of cohesion. On the one hand, respondents did identify their villages as their “community,” on the other, few reported strong feelings of trust and reciprocity between village residents. Trust was more often limited to a narrower, family context. At the community level, many respondents seemed distrustful of their leaders and often of each other; respondents reported competition for resources, extensive nepotism, division along political and socio-economic lines, and a sense that people would not come to each other’s aid, despite examples of the opposite (e.g. donating money to families following a death). One respondent said:

“There are two groups in our community one of these groups is against the ruling Party and the other group stands with the ruling party…. We are not united or one community.” (Interview A)

A number of respondents noted that help with problems should not be expected from fellow villagers, particularly those who have resources or are in distant parts of the village. Sentiments from FGDs in two communities suggest that people who have resources would not stand up for those who do not; at the same time those with resources were few in this context. One participant gave their view on the lack of mutual community support:

“You find that there is division in the community because if only one side of the village have problems it is not easy to get help from the other community members and they won’t get support from them.” (FGD 3, Participant F)

This lack of reciprocity was also reported in the political and social fabric of the communities. People cited repeatedly that ANC (ruling party) leaders, *Indunas* and CDF members favored their friends and family when it comes to resource distribution, again raising the issue of family bonds being more central than community bonds. While one church leader spoke about reliance of churches on each other, a focus group participant reported that such shared social responsibility was not, in fact, his experience.

“They [religious networks] don’t work together because today in many churches people look which kind of church you are attending. If there is bread they only share with the people from their church. They can’t call me even if they know that I have an experience for any kind of work - they won’t come to me…” (FGD 4, Participant A)

At the same time, there were reports of groups who do trust and support each other, including funeral societies and women’s savings clubs. A sense of shared responsibility was not always common around social justice, goods or services, but social needs in the face of hardship were understood to be community responsibilities and did bring about narratives of cohesion and support. In every community respondents gave the example of villagers contributing money to families following a death. In a number of interviews people also spoke of collective vigilantism as an example of villagers moving outside of formal structures and taking responsibility for criminal justice. Members of the community taking the law into their own hands by intervening to protect the community on the behalf of the common good may represent a form of collective social control or collective efficacy [41]. 

## Discussion

We set out to conceptualize the domains of community mobilization (CM) and to examine their relevancy in a rural South African community in order to guide the design of a mobilizing program intervening on intersections of HIV and gender norms. We hypothesized six domains of CM based on synergies between social sciences literature and found these domains extremely helpful as an organizational tool to conceptualize and explore CM. Examining these domains in qualitative data demonstrated that proposed domains tapped into theoretically consistent constructs that comprised relevant components of mobilization processes in rural South Africa. For example, the need for promotion of critical consciousness and fortification of community dialogue to collectively question and resolve challenges was clearly paramount to making change. The need for responsive and trusted leadership was also reported as an integral part of making change in the participant interviews. Collective action was unquestionably a mainstay of local advocacy for change, and, as in community empowerment models, was alluded to as both an integral part of the process and an outcome in and of itself [42]. However, two domains appeared to operate differently than originally hypothesized: principally organizations and networks and, to a lesser extent, social cohesion. 

Least defined in this context was the domain of organizations and networks, also understood as mobilizing structures, or vehicles “through which people mobilize and engage in collective action” [32]. In the West, these structures are envisaged as formal organizations and coalitions which can act together to provide human and material resources and dissemination networks. In rural South Africa, there were few formal organizations. Instead, the community structure centered around family-based informal networks. The lack of formal organizations could be the result of intense competition for resources, which can diminish coalition-building in the absence of a major threat (e.g. Apartheid). In the post-Apartheid era, the proliferation of small churches in this area did not translate into a formalized network (in contrast to urban settings). Additionally, the lack of formal Community Based Organizations (CBOs) could be a consequence of the structured community participation system implemented post-apartheid: structures like the CDF were established to offer a forum for input into community governance, deterring establishment of competing community interest organizations [43]. In this context, the informal and family-based networks that participants accessed for social support represent a different form of mobilizing structures that could prove strong building blocks for mobilizing; these networks also circumvent the problem of formalization of networks – which can lead to professionalization and isolation from the popular base [44]. At the same time, as compared to more formal networks, the extent to which family-based networks can access or build “bridging” social capital – that reaches beyond close local ties and connects communities and groups to more diverse networks [45] –is likely limited and thus may not provide the strongest vehicle as an organizational structure to achieve community mobilization. Truly, the extent to which informal networks can replace formal organizational development for mobilization in rural South Africa remains an important question and merits further research. It was a limitation in our research that we failed to capture more detailed information on how extended family networks were organized and maintained, and therefore how they might facilitate collective action. 

The idea of community cohesion or shared identity exists in some form in most of the theory we drew upon, but was less straightforward in our data. Community cohesion reflects a sense that a community is connected or bonded, or shares an identity. This idea that there is a cohesion or “glue” that holds people together is most often operationalized in community health promotion as shared trust and expectation to help one another, but this trust does not necessarily require close social ties [41,46]. In one South African community mobilizing project, limited community cohesion and weakened trust within the community and across community groups led to a failed program [47]. In our data, communities assisted one another in times of loss, but we found less evidence that they trusted extended community networks; with much more discussion around jealousy and competition for resources than the details of mutual assistance. This raises the questions of whether trust, shared expectations of reciprocity, or shared identity is the most appropriate CM domain in South Africa and whether these aspects of cohesion can be built during mobilizing initiatives. In social movements theory, whether a movement itself is built upon a collective identity and what role collective identity plays is contested. While some theorists believe that a collective identity logically precedes collective concerns or claims making [32,48], others have questioned whether social movements truly require pre-existing collective identities and postulate that identities could just as easily result from participation in movements [49]. Others believe that identity is both an input and central outcome of movements [50] or that collective identity is a process both formative to and key for the maintenance of social movements [51]. Construction of collective identity and establishing trust is likely a process in motion, iterative through the emergence and life of a community movement or mobilizing project [47]. Our data indicate that in rural South Africa, mutual assistance was a more salient concept than social identity, however, activities that seek to forge community cohesion (e.g. collective events focused on developing awareness around shared responsibility for community health and HIV prevention) may very well build both a sense of reciprocity and shared identity, as has been evidenced by cohesion building work with sex workers [52]. 

### Implications for future mobilizing work

There are several implications for the work planned as part of the HIV prevention trial in South Africa that are also applicable more broadly to mobilizing interventions. Most critically, intervention design can and likely should be focused as much on addressing the named mobilization domains as on the content objectives (e.g. gender norms and HIV prevention). Honing in on six identified CM domains to guide mobilization activities encourages the broadening of programmatic strategies beyond typical systems to deliver messages, such as peer education and social marketing. For example, the focus on CM domains in the HIV prevention trial now underway in South Africa has engendered building volunteer networks (CATs) to bolster organizational structure and leadership; a focus on generating opportunities for community dialogue outside of formal mechanisms (i.e. community street theatre, murals) to encourage critical consciousness; and the refinement of messaging to reframe HIV as a community concern. Meetings and training workshops with leadership are meant to instill responsibility for HIV prevention among leaders and to engage political structures in the intervention (which could also lend some resources and legitimacy) and simultaneously build new leadership in HIV and gender by recruiting community members to join volunteer Community Action Teams (CATs). For our trial, the planned intervention activities have been designed to map onto content objectives as well as mobilization objectives – all activities are cross-purpose. 

Our data also revealed that mobilizing around HIV in rural sub-Saharan Africa will require efforts to re-frame HIV as a community concern as opposed to an individual problem. In successful grass-roots mobilizing efforts for HIV/AIDS prevention, particularly that of the gay community in San Francisco in the 80’s, the understanding that HIV was lethal to an entire community was a huge impetus to address both HIV prevention and discrimination simultaneously[53]. The result was a flourishing of organizations and highly vocal advocacy campaigns – a truly collective effort to salvage the hard fought gains in gay community identity[54]. How to translate such communal urgency around HIV in rural Africa is largely unknown: despite the devastation that has already taken place, villagers did not envision HIV as something to unite them in action. Meanwhile, in the HIV prevention world, most social marketing and messaging programs have focused on condom use and partner reduction (targeting both individuals’ behaviors and social norms) but have not engaged in messaging to put a collective framing on the HIV epidemic. Our mobilization team has been engaged in ongoing efforts to design activities and messages that frame HIV as a collective problem, which we believe is needed if mobilization is to truly effect HIV prevention. 

Another important implication for mobilizing programs is how to promote leadership for HIV prevention in a setting where formal leadership is often concentrated and powerful. Partnering with and bolstering current leadership could re-enforce or entrench existing power relations, though empowerment of new leaders through project activities could also engender conflict with the current leadership. In addition, the mobilization work, specifically developing critical consciousness, could result in some challenges to local leadership in terms of calls for more transparency and accountability. Our mobilization team is attempting to manage this with a two pronged approach. First, we hold monthly leadership meetings in each village to engage current leadership in discussions about gender and HIV and ways they can support project activities. At the same time, we have set up a central intervention project office and are establishing and training community action teams (CATs) in every village in order to create more formal leadership networks for HIV and gender-related work. Because the issue of HIV and gender norms lies comfortably outside the range of current leaderships’ priorities, we hope that generating new leadership in this area will not cause tension. That said, leadership has become contested in other mobilizing projects when groups struggle over control of project financial resources [18,47]. 

### Implications for research

We have hypothesized that there are six domains necessary to building a successful mobilization program. In the coming years of the trial we aim to rigorously validate this conceptual model through monitoring change in the domains and changes in gender norms and HIV prevention behaviors over time. The longitudinal follow-up of the intervention villages will permit us to better understand whether these domains are developed simultaneously, whether some components necessarily precede others, whether all domains are critical to achieve improved outcomes (more equitable gender norms and prevention behaviors), and even how much change in any given domain might result in health impacts. This analysis requires measures of each domain; to the best of our knowledge, no such comprehensive measure exists [20,55]. Investigators have operationalized combination constructs, like “community readiness” or “capacity for change,”[56] indicators built on much of the same theory culled from the literature reviewed above. More recently the Gates funded Avahan initiative in India has produced a Community Ownership and Preparedness Index (COPI) used to assess the strength of organizational capacity among partnering CBOs, incorporating aspects of leadership and organizational networks [57]. Current measures typically target a limited number of the domains or, in the case of the COPI, would be difficult to administer outside of that specific CBO context. Based on the work presented here, we designed and are currently validating measures of community mobilization domains in order to evaluate CM efforts. The measures (forthcoming) will prove valuable tools for evaluation, both in our prevention program and for other investigators and initiatives. 

## Conclusions

This is, to our knowledge, the first attempt to synthesize relevant theory into a set of mobilization domains and explore these for the purposes of establishing theory-based HIV prevention mobilization work in rural Africa. Though community mobilization approaches for health promotion will vary across settings and issues, outlining a concrete set of domains on which to focus can facilitate and strengthen CM planning and evaluation. CM planning can improve by expanding strategies to address all six domains and program content simultaneously. At the same time, outlining CM domains contributes to the development of a more rigorous evaluation framework, which grows all the more urgent as fund allocation is increasingly limited to a strong evidence base. The domains of mobilization we developed, all of which were relevant even when adaptation was necessary, serve as a roadmap to guide CM strategies and to conduct evaluation of these approaches. We believe that CM has enormous potential to prevent the spread of HIV, to stimulate community uptake of prevention and treatment, and to change underlying social structures and power imbalances that place populations at risk. At the same time, it is critical that community capacity building and mobilizing projects should not be implemented carelessly, lest the outcome be counterproductive [58]. 
